# Lessons Learned From Managing the Planning and Implementation of Inactivated Polio Vaccine Introduction in Support of the Polio Endgame

**DOI:** 10.1093/infdis/jix185

**Published:** 2017-07-01

**Authors:** Simona Zipursky, Manish Patel, Margaret Farrell, Alejandro Ramirez Gonzalez, Tasleem Kachra, Yann Folly, Feyrouz Kurji, Chantal Laroche Veira, Emily Wootton, Lee M. Hampton

**Affiliations:** 1 World Health Organization, Geneva, Switzerland;; 2 Task Force for Global Health, Atlanta, Georgia;; 3 Programme Division, United Nations Children’s Fund, New York, New York, and; 4 Bill & Melinda Gates Foundation, Seattle, Washington;; 5 Gavi, the Vaccine Alliance, Geneva, Switzerland; and; 6 FDK Consulting, Kirkland, Washington; and; 7 Centers for Disease Control and Prevention, Atlanta, Georgia

**Keywords:** polio, polio eradication, endgame, routine immunization, vaccines, IPV, vaccine supply, vaccine shortage, vaccine introduction, inactivated polio vaccine, oral polio vaccine.

## Abstract

The Immunization Systems Management Group (IMG) was established as a time-limited entity, responsible for the management and coordination of Objective 2 of the Polio Eradication and Endgame Strategic Plan. This objective called for the introduction of at least 1 dose of inactivated polio vaccine (IPV) into the routine immunization programs of all countries using oral polio vaccine (OPV) only. Despite global vaccine shortages, which limited countries’ abilities to access IPV in a timely manner, 105 of 126 countries using OPV only introduced IPV within a 2.5-year period, making it the fastest rollout of a new vaccine in history. This achievement can be attributed to several factors, including the coordination work of the IMG; high-level engagement and advocacy across partners; the strong foundations of the Expanded Programme on Immunization at all levels; Gavi, the Vaccine Alliance’s vaccine introduction experiences and mechanisms; innovative approaches; and proactive communications. In many ways, the IMG’s work on IPV introduction can serve as a model for other vaccine introductions, especially in an accelerated context.

In May 2012 the World Health Assembly declared the completion of poliovirus eradication to be a programmatic emergency for global public health and called for a comprehensive polio endgame strategy [1]. In response, the Polio Eradication and Endgame Strategic Plan 2013–2018 (the Endgame) was developed [2]. The plan outlined a comprehensive approach for completing eradication, including the elimination of all polio disease (both wild and vaccine-related). Objective 2 of the plan called on countries to (1) introduce at least 1 dose of inactivated polio vaccine (IPV) into routine immunization (RI) schedules; (2) withdraw oral polio vaccine (OPV) in a phased manner, starting with type 2–containing OPV; and (3) strengthen RI in the 10 focus countries with the largest numbers of GPEI funded staff and assets (Afghanistan, Angola, Chad, Democratic Republic of the Congo, Ethiopia, India, Nigeria, Pakistan, Somalia, and South Sudan). The plan aimed for all type 2–containing OPVs to be withdrawn by mid-2016 and for all countries to introduce IPV prior to that withdrawal, ideally by 2015.

Introducing 1 dose of IPV into the RI schedules of all 126 countries not using IPV as of 1 January 2013 was a critical step to manage risks associated with withdrawal of the type 2 component of trivalent OPV. Once OPV type 2 was withdrawn globally, IPV’s role shifted to focus on reducing any immunity gaps by priming populations against type 2 poliovirus, incase it is reintroduced. A region where people are immunized with IPV would have a lower risk of reemergence or reintroduction of wild or vaccine-derived type 2 poliovirus. In addition, should monovalent OPV type 2 be needed to control an outbreak, those primed with IPV would be expected to have a better immune response, thus facilitating outbreak control and interruption of polio transmission. By the end of 2016, 105 of those 126 (83%) countries had introduced IPV (Figure 1). Although the earlier goal of completing global IPV introduction by the end of 2015 was not met, largely due to unexpected IPV supply limitations, IPV was still introduced in more countries between 2013 and 2016 than between its initial rollout in the United States in 1955 and 2012 [3].

**Figure 1.**
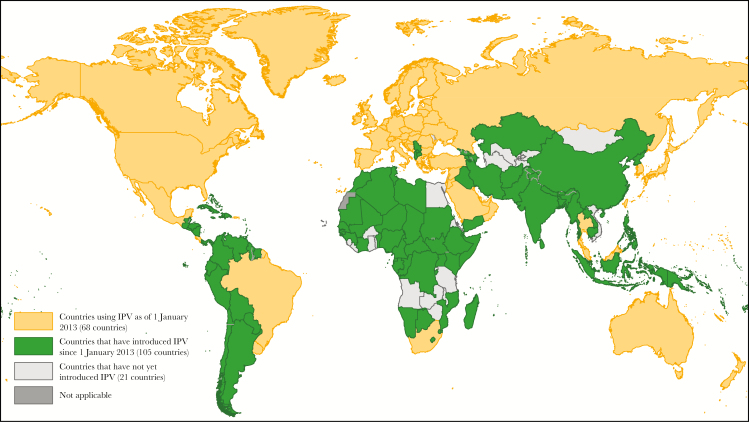
Introduction of inactivated polio vaccine (IPV) by country as of December 2016. Data source: World Health Organization Immunization, Vaccines, and Biologicals database, as of 31 December 2016.

There have been many efforts to accelerate the introduction of new vaccines globally since 2000 [4], but IPV was introduced by more countries in a 3-year period than any other vaccine over the same period of time (Figure 2) [5, 6]. Furthermore, in contrast to vaccines intended only for specific regions, such as Japanese encephalitis, yellow fever, or serogroup A meningococcal conjugate vaccine, IPV introduction targeted countries from all regions of the world. Although countries’ incomes have often greatly influenced decisions on when to introduce new vaccines [7], by 2015 all countries not using IPV had committed to introducing IPV regardless of their income status. Multiple factors led to this unusually rapid global vaccine introduction surge, including the work of the Global Polio Eradication Initiative (GPEI) Immunization Systems Management Group (IMG).

**Figure 2.**
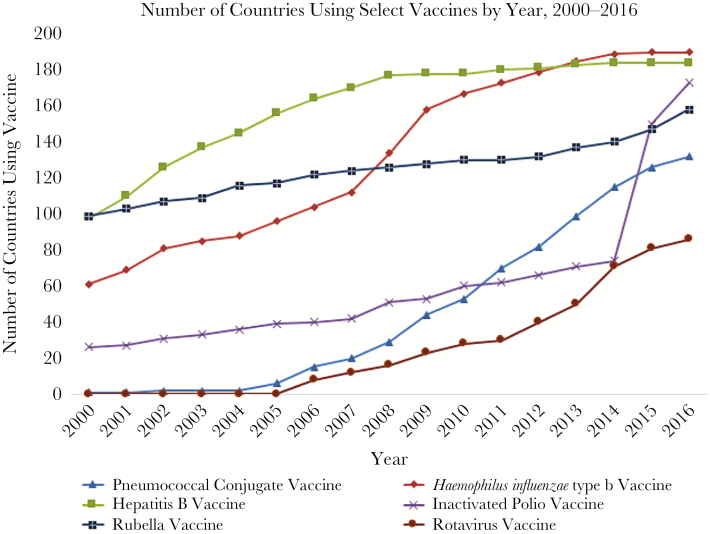
Number of countries using selected vaccines by year, 2000–2016. Data source: World Health Organization database of year of introduction of selected vaccines.

## THE IMMUNIZATION SYSTEMS MANAGEMENT GROUP

The IMG was established as a time-limited entity, responsible for the management and coordination of GPEI partners’ activities to achieve Objective 2 of the Endgame. Comprising 2 members from each GPEI partner agency (the United Nations Children’s Fund [UNICEF], the World Health Organization [WHO], the US Centers for Disease Control and Prevention [CDC], Rotary International, and the Bill & Melinda Gates Foundation) as well as Gavi, the Vaccine Alliance, the IMG was jointly chaired by WHO and UNICEF, with dedicated secretariat support at WHO. The leadership of WHO and UNICEF in chairing the IMG and maintaining its smooth operations proved to be critical to the IMG’s momentum.

The IMG had 5 subgroups: (1) the Implementation Subgroup, which oversaw IPV introduction and trivalent OPV (tOPV) withdrawal, as well as management of IPV supply; (2) the Communications Subgroup, which developed materials to support Objective 2 implementation as well as regular communication and updates to countries; (3) the Regulatory Subgroup, which addressed regulatory issues in support of IPV introduction in the 126 countries not using IPV as of 1 January 2013, as well as regulatory issues on developing pathways for the use of bivalent OPV (bOPV) in RI; (4) the Finance Subgroup, which evaluated the resources required to achieve Objective 2; and (5) the RI Subgroup, which provided advice and some funding for improving RI in 10 GPEI focus countries with the support of GPEI-funded staff and assets in those countries. These 10 countries prioritized as they had significant GPEI funded assets and staff that could be utilized to support RI, as well as having low RI coverage, putting them at risk for future polio outbreaks.

The IMG met by teleconference twice per month and in person up to twice per year. Calls were attended not only by the IMG core members but by the subgroup chairs, regional representatives, and other interested colleagues. The in-person meetings were important opportunities to review progress, share success stories, build on the momentum created, and agree on how to address challenges to moving forward.

A critical overarching factor in successful planning for IPV introduction was the broad, functional partnership that was established among the 6 IMG partner organizations. Each organization determined its level of engagement with the IMG subgroups on the basis of its specific interests and skills. This flexibility enabled each partner to contribute as needed its technical, operational, or other expertise to the planning process. Non-IMG partners were also invited to join the subgroups based on their functional experience and expertise. Critically, colleagues from WHO and UNICEF regional offices were members of each subgroup.

## STRATEGIES AND ENABLING FACTORS FOR IPV INTRODUCTION

### High-Level Engagement

IPV introduction timelines were set by the Strategic Advisory Group of Experts on Immunization (SAGE) and by the World Health Assembly. Working to meet timelines for IPV introduction set by these groups established a reputable base from which the IMG could begin its engagement with countries.

The IMG recognized early on the importance of having high-level advocates for IPV introduction. Briefings were organized in 2013 for the WHO heads of country offices in the most at-risk countries. Additionally, IPV introduction was addressed in WHO regional meetings attended by the ministers of health of all countries in each WHO region in 2013, and again as needed in 2014. To follow up, a technical briefing session on the importance of IPV introduction was held at the World Health Assembly in 2014 [8].

As appropriate, the IMG coordinated the preparation of joint letters from the WHO Director-General, the UNICEF Executive Director, and, where appropriate, the Gavi CEO, to the ministers of health to reiterate the importance of IPV introduction. The WHO and UNICEF regional directors also engaged in bilateral discussions with the ministers of health in countries of concern. The IMG also worked with national immunization technical advisory groups (NITAGs) to facilitate the decision-making process at the national level, developing a specific NITAG information kit, presentation, and decision-making materials. The IMG further collaborated with SIVAC, the NITAG creation and strengthening project, to ensure that materials were widely disseminated and that SIVAC experts could answer any questions on IPV decision making posed to them.

### Existing Knowledge of New Vaccine Introduction Within National Immunization Programs

The Expanded Programme on Immunization (EPI) was established in 1974 to fight vaccine-preventable diseases through routine immunization [9]. Over the past 15 years, the EPI has successfully introduced new vaccines such as pneumococcal conjugate vaccine, rotavirus vaccine, human papillomavirus vaccine, meningococcal conjugate vaccine, and IPV. Achieving the polio Endgame’s ambitious objective of OPV withdrawal, which began with the accelerated introduction of IPV in 126 countries and a globally synchronized switch from tOPV to bOPV [10–12], was built on the shoulders of a robust and adaptable EPI—one that had significant experience with and knowledge of new vaccine introductions, including decision making, cold chain management, vaccine procurement, and healthcare worker training.

### Adequate Financial Support

The global introduction of IPV was greatly accelerated by the financial support that was provided as required to the 126 countries that needed to introduce IPV [13]. Countries eligible or recently transitioned from Gavi support for other vaccines received financial resources from GPEI channelled through Gavi. A direct support mechanism was set up to provide catalytic support for IPV procurement and introduction costs for non-Gavi-eligible countries based on need and risk level. The direct support to non-Gavi-eligible countries is detailed in another article in this supplement [14].

### Fast-Tracking IPV Introduction Through Gavi

In 2013, GPEI requested Gavi’s support in the accelerated rollout of IPV based on Gavi’s previous success in supporting countries to introduce new vaccines, as 73 of the 126 countries that needed to introduce IPV were already receiving Gavi support for other vaccines. In June 2013, the Gavi Board endorsed Gavi’s engagement to facilitate the rollout, pending funding being provided directly by GPEI and its donors for all costs associated with IPV introduction.

Gavi provided support in 3 key ways. First, its existing infrastructure to support new vaccine introductions was adapted to support IPV. This included systems to provide countries with vaccines and injection supplies (or funding to buy locally produced vaccines, where applicable), vaccine introduction grants, and technical cooperation.

Second, Gavi made significant policy exceptions, with a focus on addressing the financial barriers given the need for rapid decision making and vaccine introduction processes within the countries. These exceptions meant that (1) all Gavi-eligible countries (including the Gavi-transitioning countries) were eligible to apply for IPV support, including those ineligible for other new vaccine support due to coverage with the third dose of diphtheria-tetanus-pertussis–containing vaccine (DTP3) <70% or default on co-financing requirements for other vaccines; (2) co-financing of IPV by the Gavi-eligible countries was encouraged, but not mandatory; (3) Gavi-transitioning countries were eligible for an IPV vaccine introduction grant (a one-time cash grant of US$0.80 per child in the birth cohort or a lump sum of US$100000 (whichever is higher) to support a share of the additional costs related to the new vaccine introduction, with any remainder necessary being funded by the government or partners); and (4) applications recommended for approval by the Independent Review Committee (IRC) were to be approved by the CEO of Gavi rather than the Gavi Board, which met only twice per year. Third, Gavi adjusted its programmatic processes, making additional investments in staff and providing dedicated budgets to its partner organizations from the GPEI funds to manage the unprecedented workload. For example, Gavi removed the requirement for countries to submit an expression of interest prior to application. Furthermore, the required application package was smaller and more flexible than applications for other vaccines, with emphasis placed on the country’s own introduction plan. Two application windows were opened specifically for IPV and 2 additional IRC sessions were held to review countries’ applications before 1 May 2015. In addition, once applications were approved, Gavi aimed to disburse vaccine introduction grants within 6 weeks of the approval, rather than 6 months before planned introduction as done for other supported vaccines.

Encouraged by these measures, countries applied for IPV support with unprecedented speed, with all expected countries applying within 14 months of the funding window being opened. (Of the 73 Gavi countries, 71 countries applied for support. Ukraine introduced in 2006 without support, and Georgia opted for a combination vaccine not supported by Gavi.) Thanks to the efforts of the partners providing technical guidance for decision making and planning, the IRC recommended the approval of all 71 country applications received. To date, Gavi has supported IPV rollout in 53 Gavi-eligible countries that were deemed higher risk and thus prioritized for IPV introduction, with the remaining 18 Gavi-eligible countries on hold due to global vaccine shortages.

### Full Engagement From the Regional and Country Offices of WHO and UNICEF

Another key factor for the success of the IMG was access to and close collaboration with the WHO and UNICEF regional offices, which provided the IMG with unique and invaluable support and expertise.

Each of the WHO and UNICEF regional offices designated a point of contact for IPV introduction. Due to the existing workload at the regional offices and the increased demand associated with IPV introduction activities, many regions contracted additional staff or consultants to serve as IPV points of contact and to assist regular immunization staff with a wide range of related tasks. The collaboration between WHO and UNICEF regional offices, which included joint work planning and activity implementation, was particularly useful.

Regional points of contact collaborated closely with their respective country offices, which were in direct contact with the government to support planning and implementation of IPV introduction. As such, the regional and country offices were well-positioned to provide thorough and timely regional- and country-level updates to the IMG and inputs into the IMG’s materials development.

The substantive engagement of regional-level colleagues in the work of the IMG became increasingly critical in the face of unexpected challenges, such as IPV supply constraints [11, 15]. Regional and country offices took on the responsibility of conveying complex and time-sensitive updates to the affected national governments clearly and in a coordinated fashion. Several articles in this supplement describe regional experiences and perspectives on IPV introduction in detail [16–19].

## SETTING THE STAGE FOR IPV INTRODUCTION: KEY OUTPUTS

### Vaccine Purchasing, Strategic Demand Forecast, and Tiering

One of the most notable challenges for the accelerated IPV introduction in the 126 countries was securing a sufficient supply of affordable IPV. To achieve this, arrangements were established among manufacturers, procurement agencies, and countries. UNICEF Supply Division played a key role in managing these relationships in its role as the vaccine procurement arm of the United Nations system, as did the Pan American Health Organization (PAHO) Revolving Fund for Vaccine Procurement, which filled that role for Latin American countries.

A crucial precondition for issuing the tender for supply of IPV for countries procuring through UNICEF and PAHO was developing global demand projections for IPV that would provide manufacturers with a comprehensive and reliable overview of the expected IPV supply requirements for the Endgame. A workgroup comprised of IMG members and Gavi secretariat staff was established to develop a strategic demand forecast for IPV. The forecast was developed in the span of 2–3 months to allow the tender to be issued by October 2013 and to also inform financial resource requirements for GPEI.

To improve operational efficiency, the IMG established a tiered strategy that grouped the 126 countries using only OPV for polio vaccination according to their risk of circulating vaccine-derived poliovirus type 2 (cVDPV2) outbreaks and importation following cessation of the type 2 component of OPV (Table 1). While all countries were planning for IPV introduction by the end of 2015, the tier criteria provided a means for directing resources, from financial support to in-country technical cooperation. The country tier assignments were reviewed biannually to capture any changes to country contexts. Although not designed for this purpose, the tiering also helped to prioritize and direct IPV supply when global supply constraints occurred in subsequent years.

**Table 1.** Summary Definitions of Risk Tiers for Inactivated Polio Vaccine Introduction Based on Risk of Circulating Vaccine-Derived Poliovirus Outbreaks and Importations Following Cessation of the Type 2 Component of Oral Polio Vaccine

Tier 1	WPV-endemic countries OR countries that have reported a cVDPV2 since 2000
Tier 2	Countries that have reported a cVDPV1/cVDPV3 since 2000 OR large/medium^a^-sized countries with DTP3 coverage <80% in 2009, 2010, and 2011 as per WUENIC, and reviewed annually for any revisions needed to the tiers
Tier 3	Large/medium countries adjacent to Tier 1 countries that reported WPV since 2003 OR countries that have experienced a WPV importation since 2011
Tier 4	All other remaining countries using oral polio vaccine

Abbreviations: cVDPV, circulating vaccine-derived poliovirus; DTP, diphtheria-tetanus-pertussis vaccine; WPV, wild poliovirus; WUENIC, World Health Organization/United Nations Children’s Fund Estimates of national immunization coverage.

^a^Small refers to live births <20000; medium, live births 20000–1000000; large, live births >1000000.

### Implementing a System for Tracking Progress

Given the importance of tracking progress in the planning and implementation of IPV introduction, the IMG established a centralized monitoring system early on. In the interest of efficiency and ease of access, the IMG employed the existing WHO Immunization Repository (accessible at https://extranet.who.int/immunization_repository/), an online portal accessible to all GPEI core agencies as well as Gavi that stores country-level information on immunization.

After an initial assessment of data elements available within the Repository, an IMG working group developed an IPV-specific set of indicators that tracked progress associated with government commitment, IPV introduction plans, introduction timeframe, vaccine procurement and shipment, regulatory issues, delays to introduction, Gavi vaccine introduction grants, training, communications, and technical cooperation (Table 2).

**Table 2.** Inactivated Polio Vaccine–Related Indicators Contained in the World Health Organization Immunization Repository

IPV-Communications plan status
IPV-Country introduction plan status
IPV-Current polio vaccine schedule
IPV-Date of first shipment of vaccine
IPV-Gavi application date
IPV-Gavi application status
IPV-Gavi vaccine introduction grant disbursement status
IPV-Has an Expression of Interest been submitted to Gavi?
IPV-Has introduction been delayed?
IPV-Introduction date
IPV-Introduction tier
IPV-IRC approval date
IPV-Joint vaccine introduction
IPV-Nationally recommended age for 1st dose
IPV-Original (baseline) introduction date
IPV-Reason(s) for delayed introduction
IPV-Registration process
IPV-TA Status: Cold chain and logistics
IPV-TA Status: Communications
IPV-TA Status: Gavi application
IPV-TA Status: Introduction plan
IPV-TA Status: Other
IPV-Training status
IPV-Vaccine introduction status
IPV-Vaccine is a third injection
IPV-Vaccine presentation (allocated)
IPV-Vaccine presentation (requested)
Is the allocated presentation of IPV licensed in country?
What will be the procurement mechanism for IPV?

Abbreviations: IPV, inactivated polio vaccine; TA, technical assistance.

The consistent engagement of and input from WHO and UNICEF regional offices, all of which were granted editorial access to the Repository, were pivotal to the accurate and real-time monitoring of country readiness and tracking of progress toward IPV introduction. The IMG assigned a central focal point who was responsible for the overall management of the IPV component of the Repository, revising indicators, monitoring and reviewing content, verifying data, and generating regular reports for the IMG and its partners.

The Repository proved a reliable and efficient platform for tracking IPV introduction progress in the 126 countries introducing IPV and provided IMG partners, policy makers, and donors with the necessary information to facilitate timely support for the Polio Endgame.

### Tailored Support to Countries

The IMG recognized the importance of a quick response to country needs to ensure that the Endgame timelines for IPV introduction could be met. A time-limited task team within the IMG collected information on various factors (Table 3) necessary for facilitating timely IPV introduction, informing partners, and targeting global and regional resources. These efforts particularly focused on countries deemed to be at highest risk of cVDPV2 outbreaks and importation postswitch to facilitate such countries’ introducing IPV before the switch.

**Table 3.** Overview of Key Global Activities and Accomplishments of Country Support

Area of Support	Activities and Accomplishments
Country assessments (Quarter 3–Quarter 4, 2013)	☑ Use of existing data sources and staff visits to identify country readiness
	• Staff visits/consultations; EPI reviews; postintroduction evaluations; annual program reviews; Gavi applications and improvement plans; WHO cold chain database; Effective Vaccine Management reports; ICC/NITAG meetings
	☑ Examples of data elements:
	• Plan for introduction (eg, start date) and, if available, introduction strategy (noting more communication from partners is required before countries have information they need to make choices)
	• Other planned introductions in 2014–2015
	• How will country make a decision about introducing IPV (eg, is endorsement by ICC/NITAG planned, will it be planned, has it occurred already)?
	• Cold chain and vaccine management gaps/needs that will not be addressed through other means (eg, Gavi’s Health Systems Strengthening) prior to IPV introduction
	• Human resources gaps or needs
	• Procurement—any obstacle that may need to be addressed, eg, customs regulation/tendering if self-procuring
	☑ Gaps identified for country decisions on IPV, cold chain, NITAG support, lack of country plans
	• Most countries did not have IPV introduction plans
	• Approximately half of the priority countries had plans for pneumococcal and rotavirus vaccine introduction
	• Cold chain gaps existed in some of the high-priority countries, motivating IMG to deploy the cold chain rapid response funds
	• Motivated development of global information material and guidance, introduction plan templates, and trained consultants
Tools, guidance documents, information materials	☑ Polio Eradication and Endgame Plan
	☑ IPV technical and general materials
	☑ SAGE policies and guidance
	☑ WHO position paper on polio (and IPV)
	☑ Technical rationale for IPV introduction
	☑ Operational manual for IPV introduction
	☑ Multiple injection materials
	☑ Dual introduction case study
	☑ Country IPV introduction case studies
	☑ Gavi documents
	☑ IPV introduction plan templates and checklists
	☑ Incremental systems cost
	☑ IPV safety documents
	☑ Workshop agenda, materials, templates (including slides, documents, checklists)
Global workshops, meetings, webinars, and early targeted country support	☑ Global consultant and staff training workshop
	☑ WHO and UNICEF regional workshops
	☑ Country workshops
	☑ Webinars targeting global partners, regional offices, and country partners
	☑ EPI manager meetings
	☑ IPV introduction plan meetings
	☑ Scientific vaccinology and infectious disease meetings
	☑ Regional and country NITAGs
	☑ Early targeted country support
	In-country cold chain assessments
	NITAG consultations
	Decision-maker and senior management consultations
	Development of IPV introduction plans
	Assistance with IPV introduction in campaigns
	Postintroduction evaluations

Abbreviations: EPI, Expanded Programme on Immunization; ICC, Interagency Coordinating Committee; IMG, Immunization Systems Management Group; IPV, inactivated polio vaccine; NITAG, National Immunization Technical Advisory Group; SAGE, Strategic Advisory Group of Experts on Immunization; UNICEF, United Nations Children’s Fund; WHO, World Health Organization.

Critical gaps were identified in cold chain readiness, and thus the IMG established a cold chain rapid response fund that supported 12 target countries to fill these gaps. The IMG partners also identified the need for decision-making support and technical guidance. This guidance included IPV introduction field guides, scientific evidence, policy statements, frequently asked questions and answers, training materials, video presentations, case studies, sample introduction plans, budget templates, and introduction checklists, along with a package of materials designed specifically for decision-making bodies.

The IMG partners developed and supported workshops and webinars that served as a launching pad for testing messages, clarifying concepts, raising awareness, and developing a cadre of highly informed individuals who subsequently provided communications, training, and other types of support worldwide. Subsequently, workshops were replicated in regions and countries, diffusing information and providing a platform for hearing country concerns and identifying countries’ needs for specific support.

The IMG engaged with countries in a variety of ways, supporting activities such as country consultations, sensitization workshops, introduction plan development, cold chain assessments, and training workshops. Early successes included providing targeted support to countries voicing early interest in IPV introduction and use of IPV in campaigns. The IMG’s success with technical cooperation was due to its broad approach to anticipating country needs, developing a host of materials, identifying and allocating resources, deploying technical experts, broadly disseminating information, and receiving feedback to modify messages and materials as needed to facilitate the timely and successful introduction of IPV worldwide.

### Implementation of the Multidose Vial Policy

When UNICEF made its initial IPV award to manufacturers in May 2014, most of the supply requirements were projected to be covered by 5-dose and 10-dose vial presentations, with only limited quantities of single-dose vials being available. At the time, it was unclear if the preservative in the multidose vials (2-phenoxyethanol) met WHO requirements for effectively preserving the vaccine for 28 days after opening. Open IPV vials therefore needed to be discarded at the end of each session or 6 hours after opening. This restriction created potential for high wastage, depending on the number of children per session.

In the absence of country-specific data, countries were advised to plan for maximum wastage values of 50% for 10-dose vials and 30% for 5-dose vials. High vaccine wastage can lead to vaccine stockouts. In addition, lower vaccine coverage may occur if health workers are reluctant to open multidose vials for only a few children per session [20].

Production delays for the 5-dose vials led to reliance on higher wastage 10-dose vials, placing further strain on the global supply of IPV. These considerations prompted WHO to conduct extensive discussions with manufacturers and regulatory authorities to request a more thorough assessment of newly generated data to explore if multidose IPV vials could be safely used beyond 6 hours after opening. Precedent existed for such an approach. In 2012, stockouts of IPV were stopped in Brazil after national regulatory authorities issued a label change permitting use of 10-dose vials for 7 days after opening on the basis of manufacturer submitted data [21].

In October 2014, the WHO prequalification team convened a scientific advisory group which reviewed the available data and confirmed that the data supported the safe use of opened multidose vials for up to 28 days in accordance with the WHO policy on multidose vials, once manufacturers placed vaccine vial monitors on the label [22]. The efforts by IMG, manufacturers, and regulatory authorities to fast-track this process while maintaining rigor eased the logistics of IPV introduction globally in an already supply-constrained atmosphere.

### Addressing Multiple Injections

The introduction of IPV meant many low- and middle-income countries were faced with the prospect of administering >2 injectable vaccines at a single visit for the first time [23, 24]. Some immunization program managers expressed concern that vaccinators and caregivers might refuse to allow children to receive >2 injectable vaccines in a single visit, resulting in lower immunization coverage. However, data from countries that had been giving ≥3 injectable vaccines at a single visit indicated that while vaccinators and caregivers might express concerns about multiple injections, in practice they were very likely to comply with national recommendations [23].

On the basis of these data, the IMG created communications and training materials to reassure immunization program staff and help train them on the administration of multiple injectable vaccines. These messages were further reinforced and supported through an evidence-based SAGE review on multiple injections [24, 25]. Ultimately, most countries did adopt schedules in which 3 injectable vaccines would be administered at a single visit, and early evaluations of these experiences have found that uptake of IPV and other vaccines was high [25–27].

## MANAGING THE UNEXPECTED CHALLENGES FACING IPV INTRODUCTION

### Coordinated Global Monitoring and Allocation of IPV Supply

While all countries committed to introduce IPV before the switch, efforts to introduce at least 1 dose of IPV in all 126 target countries by the end of 2015 were greatly complicated by supply shortfalls. These were due to vaccine production scale-up challenges faced by both of the vaccine manufacturers that supplied IPV to UNICEF supply division, the PAHO Revolving Fund for Vaccine Procurement, as well as several key countries that procure their vaccines directly from these manufacturers. There was also an increased demand for IPV for outbreak response and endemic country campaigns, which had not originally been envisaged. Outreach to other IPV manufacturers who were not supplying IPV to GPEI did not identify any additional vaccine availability. As of the end of 2016, these unexpected supply shortfalls accounted for a >40% decrease from the amount originally committed as part of the vaccine procurement tender process. This lack of supply presented a huge management and communication challenge and resulted in delays in IPV introduction.

A Supply Task Team was created as part of the IMG Implementation Subgroup to manage the available supply. Its mandate was to develop principles for allocation of the available supply using a risk-based approach that used the tiers as its starting point which was subsequently approved by the Polio Oversight Board, GPEI’s governing body. This approach prioritized the use of IPV to support eradication efforts in polio-endemic countries, followed by use of IPV in countries assessed to be highest risk of cVDPV2 outbreaks and importation postswitch (Tier 1 and 2 countries). Remaining IPV was allocated to outbreak response in nonendemic countries and introduction into routine immunization programs in lower-risk countries (Tier 3 and 4 countries). By mid-2016, this approach resulted in 20 countries having to delay introduction of IPV until at least the fourth quarter of 2017 and another 29 countries that had already introduced IPV having their next IPV supply delayed sufficiently that they were expected to have a national IPV stockout [11]. However, all countries deemed to be at the highest risk of cVDPV2 outbreaks postswitch were able to provide IPV to infants through their routine immunization programs.

The open and collaborative interaction among IMG partners was critical to handling this complex situation and ensuring that GPEI communicated the news regarding delays in IPV availability to countries with one unified voice. The IMG made the decision early on that information and updates would be shared as they became available, noting which information was not yet available and when it should be expected. This transparent approach was appreciated by partners and country-level colleagues. Communications included the issuance of joint information bulletins, synchronized emails to country offices, and commitment to uphold the agreed position of the partnership by all agencies during bilateral discussions.

### Delivering Fractional Doses of IPV Intradermally

The IMG, along with other GPEI groups, provided technical guidance to countries interested in using fractional doses of IPV delivered intradermally instead of full-dose IPV, to maximize the number of children who could be immunized given the limited supply of IPV. By February 2016, the global shortage of IPV had become severe enough to threaten the ability of India to provide IPV to children in all of its states [28]. Drawing on previous research which showed that 2 fractional doses of IPV administered intradermally, each of which uses one-fifth the volume of a full IPV dose administered intramuscularly, can elicit a stronger immune response than a single full dose of IPV administered intramuscularly while using less total IPV volume [29, 30], India decided to use intradermal fractional IPV in selected areas. Due to uncertainties about how well fractional IPV would be administered under field conditions, fractional dose IPV was initially used in Indian states considered to be at lower risk for cVDPV2 outbreaks while using full-dose IPV in the states at higher risk.

In March and April 2016, the WHO SAGE encouraged other countries to consider administering 2 doses of fractional IPV instead of a single full dose [31]. The IMG disseminated information on how countries might implement fractional IPV dosing and provided technical and logistical guidance to interested countries, including collaboration to secure syringes and injection aids for administering fractional dose IPV. As of December 2016, 1 additional country, Sri Lanka, had adopted the use of fractional dose IPV [11].

## CONCLUSIONS

The high level of commitment from national governments to introduce IPV within GPEI’s timelines shows the potential to rapidly introduce a new vaccine with the right set of support and incentives. Unfortunately, IPV introduction was delayed in many countries due to the global vaccine supply shortage. However, the efforts of the IMG to use the available supply efficiently ensured that the most children possible could be covered. The first phase of the global withdrawal of OPV, the global switch from tOPV to bOPV, went ahead in April 2016 despite the IPV shortages, with all OPV-using countries reporting that they had ceased use of tOPV by 12 May 2016 [11]. The collaboration and cooperation among IMG partners were critical to these accomplishments.

Nevertheless, a great deal of work remains if the full potential of IPV is to be realized. In addition to the need to introduce IPV in the remaining countries that were delayed due to supply constraints, and resupplying those forced into stockouts, sustained effort will be needed to ensure IPV use continues after GPEI funding ceases. The IMG’s coordination efforts, along with having access to the necessary resources, were critical to the rapid pace of IPV introduction during 2013–2016. The overall success of global IPV introduction between 2013 and 2016 suggests that it could be a viable model in many ways for efforts to introduce other vaccines globally, particularly if a vaccine needs to be rapidly introduced across a large number of countries.

## Notes

***Disclaimer.*** The findings, interpretations, and conclusions expressed in this article are those of the authors and do not necessarily reflect the policies or views of their respective organizations.

***Financial support.*** M. F., M. P., and C. L. V. were supported through a grant by the Bill & Melinda Gates Foundation (grant number OPP1095024). Gavi, to support its role in the polio eradication efforts, received funds from the UK Department for International Development, the Norwegian Agency for Development Cooperation, and the Bill & Melinda Gates Foundation, a small portion of which was used by Gavi to support Gavi secretariat staff (Y. F., E. W.).

***Supplement sponsorship.*** This work is part of a supplement coordinated by the Task Force for Global Health with funding provided by The Bill and Melinda Gates Foundation and the Centers for Disease Control and Prevention.

***Potential conflicts of interest.*** M. F., M. P., and C. L. V. have received grants from the Bill & Melinda Gates Foundation. F. K. has consulted to Bill & Melinda Gates Foundation. T. K. is employed by the Bill & Melinda Gates Foundation. All other authors report no potential conflicts of interest. All authors have submitted the ICMJE Form for Disclosure of Potential Conflicts of Interest. Conflicts that the editors consider relevant to the content of the manuscript have been disclosed.
